# Medical treatment of an unusual cerebral hydatid disease

**DOI:** 10.1186/s12879-017-2935-2

**Published:** 2018-01-05

**Authors:** Shu Chen, Ning Li, Feifei Yang, Jiqin Wu, Yuekai Hu, Shenglei Yu, Qi Chen, Xuan Wang, Xinyu Wang, Yuanyuan Liu, Jianming Zheng

**Affiliations:** Department of infectious diseases, Huashan Hospital, Fudan University, Shanghai, China

**Keywords:** Hydatid disease, Brain, Albendazole

## Abstract

**Background:**

Hydatid disease is a worldwide zoonosis produced by the larval stage of cestodes of the *Echinococcus* genus. Hydatid disease primarily involves the liver and lungs. The brain is involved in less than 2% of cases. Surgery has long been the only choice for the treatment, but chemotherapy has been successfully replaced surgery in some special cases.

**Case presentation:**

We report a rare hydatid disease case which presented with multiple lesions in right frontal lobe, an uncommon site, and in the liver and lungs. A 28-year-old woman presented with 6 months history of recurrent convulsion. Cranial magnetic resonance imaging found multiple lesions in right frontal lobe, so she was hospitalized for surgical treatment and received sodium valproate by oral for controlling epilepsy. Before the operation, other lesions were found in the liver and lungs by computerized tomography scan. There were multiple pulmonary nodules near the pleura and large cyst in the liver. The pathology of liver showed that it may be a hydatid disease. Then, positive serum antibodies for echinococcus antigen further confirmed our diagnosis. Since her central nerve system was involved, she received four pills (800 mg, about 17 mg/kg/day) albendazole treatment for 18 months without operation. Her symptoms abated and a follow-up magnetic resonance imaging showed that the lesion had obviously diminished after treatment. She was recurrence free 2 years after we stopped albendazole treatment.

**Conclusions:**

This case reveals an uncommon pattern of intracranial hydatid disease. Albendazole can be beneficial for some inoperable cerebral hydatid disease patients.

## Background

Hydatid disease is a worldwide zoonosis produced by the larval stage of cestodes of the *Echinococcus* genus. The disease is endemic in many parts of the world, particularly in the Middle East, Australia, New Zealand, South America and central and south Europe [1]. Hydatid disease primarily involves the liver and lungs. The brain is involved in less than 2% of cases [2, 3].

## Case presentation

**Fig. 1:**
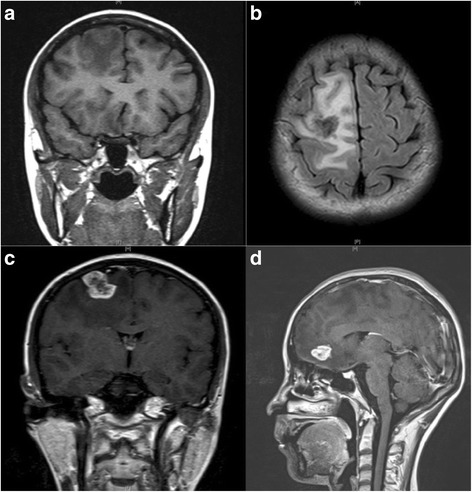
MRI scan of brain: **a** Coronal view of T1 W flair. The lesion was with low signal intensity on T1 W flair image. **b** Axial view of T2 W flair. The lesion was with low signal intensity and edema zone was seen around it on T2 W flair image. **c** Coronal view of T1 W flair + C. The lesion was with minimal rim enhancement. **d** Sagittal view of T1 W flair + C. The lesion was with minimal rim enhancement

**Fig. 2:**
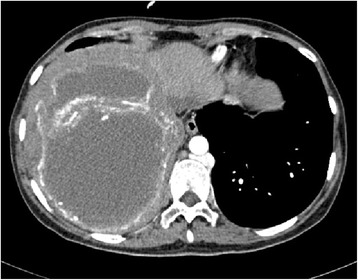
CT scan of liver. CT scan revealed multiple large daughter cysts with peripheral focal areas of calcification

**Fig. 3:**
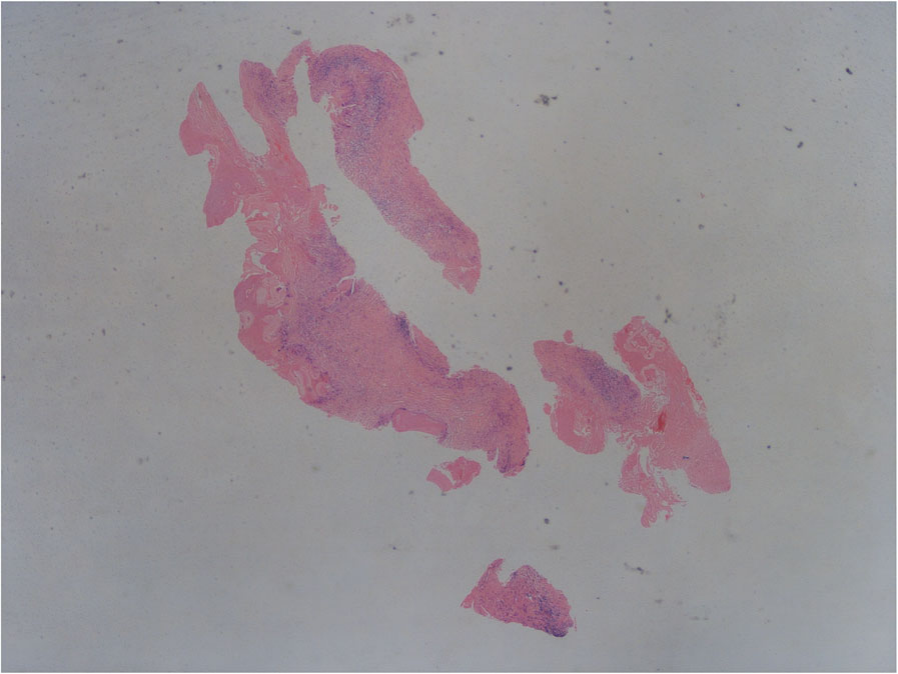
The pathology of liver biopsy. Histologic view of the liver (hematoxylin-eosin, original magnification ×100). That showed Granulomatous inflammation with necrosis and foreign body giant cell reactions

**Fig. 4:**
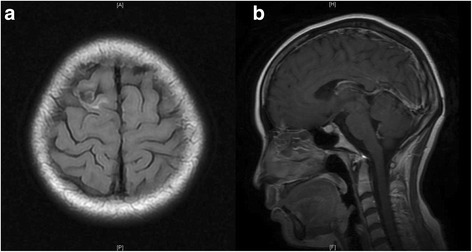
MRI scan of brain after albendazole treatment: **a** Axial view of T2 W flair. The lesion had obviously diminished after treatment. **b** Sagittal view of T1 W flair + C. The lesion had obviously diminished and was without enhancement

A 28-year-old woman presented with 6 months history of recurrent convulsion. Cranial magnetic resonance imaging (MRI) found multiple lesions in right frontal lobe which were thought to be the cerebral metastasis (Fig. 1), so she was hospitalized for surgical treatment and received sodium valproate by oral for controlling epilepsy. On MRI, the lesions were low signal intensity on both T1-weighted and T2-weighted images, had edema zone on T2-weighted images, and were with minimal rim enhancement. Before the operation, other lesions were found in the liver and lungs by computerized tomography (CT) scan (Fig. 2). There were multiple pulmonary nodules near the pleura and large cyst in the liver. The liver biopsy was done and the pathology showed it may be a hydatid disease (Fig. 3). Then, positive serum antibodies for echinococcus antigen further confirmed our diagnosis. Since her central nerve system was involved, she received four pills (800 mg, about 17 mg/kg/day) albendazole treatment for 18 months without operation, and had no side effects. Her symptoms abated and a follow-up cranial MRI showed that the lesions had obviously diminished after treatment (Fig. 4). She was recurrence free 2 years after we stopped albendazole treatment.

## Discussion and Conclusions

The liver involvement is the most frequent manifestations of echinococcosis, the lungs involvement is the second most common manifestations, but the brain involvement is rare [4]. The parasites located in the brain can cause serious neurologic symptoms, including paralysis and seizures. Cerebral hydatid disease is usually diagnosed during childhood and is often solitary [4, 5]. Although hydatid disease may be located anywhere in the brain, it is most frequently found in both hemispheres, particularly in the middle cerebral artery territory with the parietal lobe being the most common site [2, 5]. The differential diagnosis of intracranial multiple lesions is wide, including cerebral abscesses and tumors. However, the mass effect is prominent, surrounding edema and obvious enhancement are usually seen in abscesses and tumor. The lesions are usually low signal intensity in hydatid disease, unlike those are high signal intensity in abscesses and tumor on T2-weighted images.

To our knowledge, only three cases involving frontal lobe with albendazole treatment has been reported up to now, but none of them was presented with multiple lesions and cured only using albendazole without operation in an adult like this case [6–8]. This patient was diagnosed when she was adult and with multiple lesions in right frontal lobe, an uncommon site of central nerve system. Surgery has long been the only choice for the treatment. However, in inoperable disease, chemotherapy with anthelmintic medication is the only treatment shown to be potentially effective, but is usually palliative [9, 10]. Although surgery remains the main treatment option, percutaneous procedures and chemotherapy have been successfully replaced surgery in some selected cases [10].

Compared to mebendazole, albendazole is a better treatment option for hydatid disease, because of its better resorption [11]. Albendazole can be beneficial for inoperable patients, with multiple cysts and in the lungs and liver hydatid disease [10, 12]. It can be administered orally at a dosage of 10 to 15 mg/kg/day, and administration should be continuous without interruptions. However, the optimal dosage of albendazole and optimal duration of treatment are still unknown. Since her central nerve system was involved, we planned to use albendazole treatment at a high dosage, 15 mg/kg/day. Because her body weight was 47 kg, she need 705 mg albendazole per day. One pill of albendazole was 200 mg, so we gave her four pills per day, about 17 mg/kg/day. Fortunately, she had no side effects during the treatment, although she used a little higher dosage of albendazole. Finally, after we stopped albendazole treatment, we followed up this patient 2 years and she was still recurrence free.

Infection of the central nervous system, caused by *Echinococcus* genus, is rare, and the mainstay of treatment is surgical excision of the intracranial lesions [13]. Fortunately, our patient was successfully cured by albendazole treatment without operation.

## Abbreviations

CT: Computerized tomography
MRI: Magnetic resonance imaging
